# Exploring the recent trends in perturbing the cellular signaling pathways in cancer by natural products

**DOI:** 10.3389/fphar.2022.950109

**Published:** 2022-09-08

**Authors:** Md. Mominur Rahman, Md. Taslim Sarker, Mst. Afroza Alam Tumpa, Md. Yamin, Tamanna Islam, Moon Nyeo Park, Md. Rezaul Islam, Abdur Rauf, Rohit Sharma, Simona Cavalu, Bonglee Kim

**Affiliations:** ^1^ Department of Pharmacy, Faculty of Allied Health Sciences, Daffodil International University, Dhaka, Bangladesh; ^2^ Department of Pathology, College of Korean Medicine, Kyung Hee University, Seoul, South Korea; ^3^ Department of Chemistry, University of Swabi, Swabi, Anbar, Pakistan; ^4^ Department of Rasa Shastra and Bhaishajya Kalpana, Faculty of Ayurveda, Institute of Medical Sciences, Banaras Hindu University, Varanasi, Uttar Pradesh, India; ^5^ Faculty of Medicine and Pharmacy, University of Oradea, Oradea, Romania

**Keywords:** cancer, natural compounds, therapeutic efficacy, reactive oxygen species, metastasis

## Abstract

Cancer is commonly thought to be the product of irregular cell division. According to the World Health Organization (WHO), cancer is the major cause of death globally. Nature offers an abundant supply of bioactive compounds with high therapeutic efficacy. Anticancer effects have been studied in a variety of phytochemicals found in nature. When Food and Drug Administration (FDA)-approved anticancer drugs are combined with natural compounds, the effectiveness improves. Several agents have already progressed to clinical trials based on these promising results of natural compounds against various cancer forms. Natural compounds prevent cancer cell proliferation, development, and metastasis by inducing cell cycle arrest, activating intrinsic and extrinsic apoptosis pathways, generating reactive oxygen species (ROS), and down-regulating activated signaling pathways. These natural chemicals are known to affect numerous important cellular signaling pathways, such as NF-B, MAPK, Wnt, Notch, Akt, p53, AR, ER, and many others, to cause cell death signals and induce apoptosis in pre-cancerous or cancer cells without harming normal cells. As a result, non-toxic “natural drugs” taken from nature’s bounty could be effective for the prevention of tumor progression and/or therapy of human malignancies, either alone or in combination with conventional treatments. Natural compounds have also been shown in preclinical studies to improve the sensitivity of resistant cancers to currently available chemotherapy agents. To summarize, preclinical and clinical findings against cancer indicate that natural-sourced compounds have promising anticancer efficacy. The vital purpose of these studies is to target cellular signaling pathways in cancer by natural compounds.

## Introduction

Cellular signaling is a complicated signaling network that governs and organizes cells’ key biological processes. Signaling cascades are three-dimensional protein pathways that interact with one another in a specific cell site (Shaw and Filbert, 2009). Cancer cells regularly exhibit changes in several cellular signaling pathways as a result of the complex transmission of cell signaling. This could explain why specific inhibitors that target a single pathway have so often failed to treat cancer. In cancer cells, the cell cycle and apoptosis regulatory systems almost always fail, resulting in uncontrolled cell proliferation and tumor formation [1]. NF-B, Akt, MAPK, Wnt, Notch, p53, AR, and ER, among others, have been discovered as cellular signaling pathways that influence cell growth and death. All of these signaling pathways are damaged in cancer cells, which promotes the growth of cancer cells and inhibits apoptosis (Karin et al., 2002; Martin, 2003; Sebolt-Leopold and Herrera, 2004; Stiewe, 2007; Klaus and Birchmeier, 2008). As a result, in order to effectively eradicate cancer cells, it is important to create a technology that can concurrently target many biological signaling pathways (Sarkar et al., 2009).

Chronological diseases that severely endanger human life are cancers. Many treatments have been developed for cancer care, involving surgery, radiotherapy, targeted therapy, and chemotherapy. The occurrence pace of malignancy has been steady in women. It has decreased marginally in men over the past decade (2006–2015) due to all these therapies, and the cancer mortality rate (2007–2016) has also decreased (Siegel et al., 2019). In 2018, cancer caused an expected 9.6 million deaths, and cancer is predicted to be the primary source of death worldwide in the 21st century (Bray et al., 2018; Rahman et al., 2022a; Rahman et al., 2022b). Chemoprevention is a somewhat expensive option for treating cancer. The idea is gaining popularity because it is more affordable and efficient. Chemoprevention involves the administration of one or more naturally occurring and synthetic agents. It can prevent, slow down, or reverse the initiation and progression of the disease (Haddad and Shin, 2009). Berenblum pioneered chemoprevention in the 1920s, and Sporn’s work in the 1970s brought it back into the mainstream of cancer research (Amin et al., 2009).

Consequently, in addition to cancer treatment, malignancy counteraction stays an inventive region of anticancer science. Because of increased studies, the components of variant sign transduction pathways in malignant growth and the effects of these pathways on apoptosis, tumorigenesis, and metastasis have been progressively exposed (Lezhnina et al., 2014). Traditional cancer treatment strategies, however, are only successful for certain malignant tumors (Sun, 2015). Unscheduled and unregulated cell proliferation characterizes it in the cell spectrum. Cancer incidence in developed nations has been tumor forms associated with viral, genetic mutations, and bacterial infection have prevailed (Jemal et al., 2010). Metastasis, heterogeneity, recurrence, resistance to radiotherapy and chemotherapy, and shirking of immunological surveillance are the key reasons cancer treatment has failed (Batlle and Clevers, 2017). In its growth and the progression of the disease, cancer has a high occurrence and a long dormancy time. Several risk factors for cancer development are identified, including geographic region, age, and race (Wiart, 2007). The revelation of various biomarkers related to antibiotic development, and even as epidermal growth factor receptor (EGFR), cyclo-oxygenase-2 (COX-2), and Ras, as well as the invention of Novel tailored inhibitors for such biomarkers, has opened up new avenues for chemoprevention (Arber et al., 2009; Toshiya et al., 2012). Numerous targeted medicines have since been found, including the COX-2 inhibitors celecoxib and rofecoxib, the EGFR inhibitors erlotinib and gefitinib, and farnesyltransferase inhibitors (Deng et al., 2019; Forouzanfar and Mousavi, 2020).

Macro autophagy, or autophagy for short, is a highly conserved stress repercussion and degradation process. Stresses such as hunger trigger autophagy, which results in double-membrane autophagosomes capturing intracellular proteins and organelles (Deng et al., 2019). Cargos (protein clusters and malfunctioning mitochondria) will be carried to lysosomes for breakdown, allowing biomolecules to be recycled and lifespan to be maintained (Nawaz et al., 2020). Autophagy governs several cellular activities, including attribute monitoring and the destruction of defective proteins and organelles, in addition to its functions in energy homeostasis (Singh et al., 2017a). As a result, defective autophagy can play a role in developing various diseases, notably cancer, neurological sickness, and immune disorders (Dikic and Elazar, 2018; Wang et al., 2019; Rahman et al., 2021a; Rahman et al., 2021b; Rahman et al., 2021c; Islam et al., 2021; Rauf et al., 2021; Bhattacharya et al., 2022; Rahman et al., 2022c; Rahman et al., 2022d). Several bits of research point to a relationship between nutrition and cancer therapy efficacy. Appropriate utilization of phytochemicals arising from dietary and medicinal herbs has been shown to lower cancer fatality by influencing the stimulation of numerous oncogenic molecules (Singletary and Milner, 2008; Hsieh et al., 2015).

**FIGURE 1:**
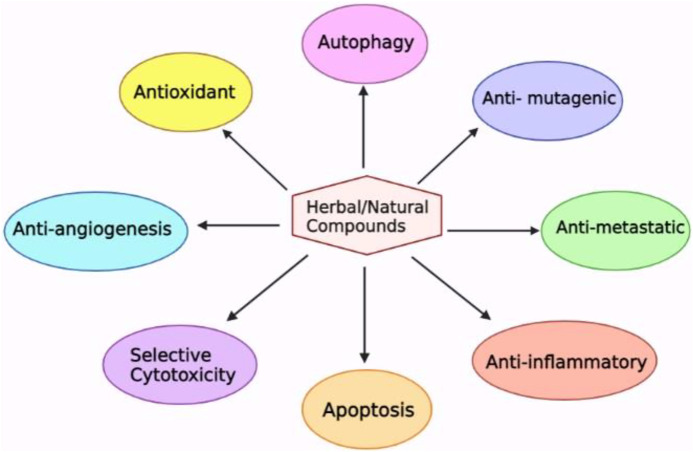
Aspects of herbal substances that contribute to their chemotherapeutic action (Laskar et al., 2020).

Natural cancer chemoprevention, particularly phytochemicals, vitamins, and minerals has emerged as a viable and practical way to reduce cancer’s impact and is gaining traction as a safe and cost-effective substitute to anticancer therapy. Contrary to pharmaceutical medications, which are mono-target molecules, herbal remedies have multitarget compounds that can control the initiation and spread of cancer (Deeb et al., 2007; Shu et al., 2010; Khan et al., 2015). Herbal substances exhibit several anti-cancer properties, particularly anti-inflammatory, anti-mutagenic, anti-oxidative, and apoptosis-inducing properties, which may aid in preventing cancer growth in its initial phase **(** Figure 1 **)**. By suppressing the cell cycle, inducing apoptosis, controlling the metabolism of carcinogens and the expression of oncogenes, restricting cell adhesion, multiplication, and migratory, and blocking signaling pathways that are crucial for cancer development, dietary ingestion of a sufficient amount of these herbal supplements may aid in the preventive measures and treatment of cancer (Huang et al., 2009). A total of 136 anticancer medications were introduced for usage between 1981 and 2014, and about 83 percent of them were either herbal substances or their derivatives (Amaral et al., 2019).

In various studies in both *in vitro* and *in vivo* settings, the prevention of cancer by natural compounds, particularly phytochemicals, minerals, and vitamins, has shown encouraging outcomes against different malignancies (Manson, 2003). Raw materials have a rich and long tradition when it comes to producing bioactive chemicals. As a significant novel source with a wide variety of pharmaceutical promise, herbal medicines are used to cure human diseases, involving nearly all forms of cancer (Christen and Cuendet, 2012; Rahman et al., 2022e; Rahman et al., 2022f). The inclusion at epigenetic, genomic, molecular, and cellular levels of several factors influencing the developmental stages of cancer opens up immense opportunities to disrupt and reverse the onset and development of the disorders. It offers various targets for scientists and researchers to avoid cancer growth through physiological and pharmacological mechanisms (Cragg and Newman, 2005; Chopra et al., 2022; Rahman et al., 2022g; Rahman et al., 2022h; Rahman et al., 2022I; Rahman et al., 2022J; Rahman et al., 2022K; Rauf et al., 2022). This article will focus on advances in understanding the signaling pathways of cancer by natural compounds. According to recent findings from an increasing number of studies on these natural products, these natural compounds exhibit their pleiotropic effects on cancer cells by targeting numerous cellular signaling pathways, such as NF-B, MAPK, Wnt, Akt, Notch, p53, AR, and ER pathways. This suggests that these natural compounds might be useful alone or in combination with conventional therapeutic agents for the prevention and/or treatment of tumor development. This article summarizes the functions of many of these signaling pathways. It also reflects the various natural-sourced compounds and their associated mode of action in various cancer lines with chemical structure. The newly found anticancer drug (dostarlimub) and its mode of action are also briefly discussed in this article. This article is unique due to the brief overview of numerous cancers signaling pathways, natural compounds and their modes of action, and recently identified anticancer medicines with modes of action. Overall, this article on using natural resources as sources for the development of novel anticancer drugs abundantly supports the idea that natural resources remain very attractive for the discovery and manufacture of cutting-edge medical devices for the treatment of cancer patients. Phytoconstituents separation of novel compounds and their molecular and cellular analysis in *in vitro* cancer cells must come first in order to develop new drugs with improved pharmacological features regarding efficacy to target tumor cells and safety to spare adverse effects towards normal tissues.

## Cancer management signaling pathways

Cancer is the driving reason for death in developed countries, with a dynamic mechanism of improvement in pathophysiology. It causes cells to avoid homeostatic controls. Creating an effective therapeutic strategy that tackles all fanatical cancer components (Sever and Brugge, 2015). Many therapeutic strategies are currently being developed for the prevention and treatment of cancer. A small number of rectal cancer patients recently witnessed a sort of scientific miracle: after receiving an experimental medication, their cancer just disappeared. Patients took the medication dostarlimab for 6 months as part of a relatively limited trial run by specialists at the Memorial Sloan Kettering Cancer Center in New York. Every single one of their malignancies vanished as a result of the experiment. There is plenty to learn about the treatment’s mechanism of action since this trial group consisted of only 18 individuals (Guardian, 2022). GlaxoSmithKline, a pharmaceutical manufacturer, manufactured dostarlimab. The medicine cost $11,000 per dose and was administered to the individuals every three to 6 months. The medicine is referred to as a checkpoint suppressor. Cancer cells create a barrier around themselves to thwart T-cells from the body’s immune system from destroying them. This medication removes that barrier. Cancer cells are more susceptible to being eliminated by the immune system when the shield is absent. Over a follow-up term, which lasted an average of about a year, none of the individuals had a return of their disease or required additional therapy. None of the patients encountered the more severe side effects that are frequently associated with rectal cancer therapies, such as infertility, neuropathy, or sexual dysfunction, despite some of them having rashes, dermatitis, exhaustion, pruritis, or nausea (Cercek et al., 2022). The dostarlimub injection inhibits the activity of a specific protein in cancer cells, which is how it works. This aids in slowing the growth of tumors and aids the immune system’s defense against cancer cells (Adnal, 2022). The initiation of unregulated cellular proliferation and disease metastasis cancer can be separated in multiple pathological stages of a natural cell’s mutation into cancer cells due to deregulation of different unique pathways. The nuclear factor kappa B (NF-κB) has been shown in the action stage of cancer where normal cells are transformed into cancer cells, improvements in immunological and cellular pathways, and relaxation (Lin et al., 2010; Vinay et al., 2015). NF-κB is an inducer of inflammation. The cellular connections between inflammation and carcinogenesis are explored, and there is mounting evidence that NF-kB (Nuclear factor kappa B) plays a critical role in the correlation between them. The regulation of innate and adaptive immune responses that are present in inflammatory states and the integration of various stress stimuli depends on transcription factors (NF-kB) (Karin et al., 2006). It was only reasonable to anticipate a connection between NF-kB and cancer, as was initially proposed several years ago, given the understanding that inflammatory diseases are frequently related to or cause cancer (KarinM et al., 2002). Since then, a rapid accumulation of experimental data has revealed precise pathways by which NF-kB affects cancer to start, develop, and progress. The emergence of RelA/p65 cloning and sequencing immediately exposed its kindred to c-Rel and its malignant variant v-Rel, which led to the emergence of a potential link between NF-kB and cancer. Oncogenic mutations that confer transformative action on RelA, c-Rel, or other NF-kB proteins, on the other hand, were discovered to be uncommon and mostly restricted to lymphoid malignancies (Gilmore, 2003).

If inflammation and infection promote tumor growth, they must do so via altering signal transduction pathways that affect cells engaged in either cancer monitoring or malignant transformation. Unless they contain their oncogenes, poisons, or growth regulators, infectious organisms often affect the recipient by activating pattern recognition receptors (PRRs), such as components of the Toll-like receptor (TLR) family (Medzhitov, 2001). As a result of the activation of multiple signaling pathways by PRR and TLR activation, many transcription factors are targeted, which regulate genes expressing cytokines, chemokines, and enzymes that drive innate and adaptive immune responses (Karin et al., 2006). The inflammatory response is propagated and amplified by a number of these genetic variants that stimulate certain receptors; it is one aspect of the broader innate immune response. While activating multiple important signaling pathways, activation of TLRs and receptors for proinflammatory cytokines, including as TNF-a and interleukin (IL)-1, plays a crucial role in inflammation and innate immunity (Li and Verma, 2002). Uncontrolled cell multiplication is responsible for the dissatisfaction of the autophagy and apoptotic pathway. They assume that the second stage of cancer is an important aspect of sustaining stable cell endurance and eradicating mutant cells (Hassan et al., 2014; Nagelkerke et al., 2015). The metabolic pathway continues to change as a consequence of the increased energy requirements of cancer cells. Every location where RAS protein activity occurs, which is required for the signaling pathway of RAS proteins to function, represents a new stage of cancer (Galluzzi et al., 2013; Bahrami et al., 2018). Lopsided angiogenesis pathways regulate the mechanism for cancer attack and metastasis of new organs in the high-level phase of cancer (Zhao and Adjei, 2015). Table 1 describes the clinical goals of the different processes involved in cancer treatment and their therapeutic action mechanisms.

**TABLE 1:** Targets of common pathways implicated in cancer treatment with clinical action strategies (Singh and Shukla, 2018).

Pathway	Targets	Activity	Targeting mechanisms	References
Immune Pathway	Regulatory T cells (Tregs)	Reduce the immune system’s reaction.	Reducing Tregs have a suppressive effect.	Byrne et al. (2011)
	Tumor Necrosis Factor Receptor (TNFR)	T-cell activation and immune responses may be manipulated.	TNFR contact inhibition.	Du et al. (2017)
	II NK cells and M2 macrophages	Growth of a tumor	Release of cytokines is inhibited.	Singh et al. (2017b)
Metabolic pathway	HK1 and HK2	Phosphorylation of ATP	Silencing of HK2 and HK1.	Singh et al. (2015); Gupta et al. (2017)
	3BP	ATP supply is being reduced.	Increasing the supply of 3BP.	Shoshan, (2012)
	GAPDH	For energy, break down glucose.	GAPDH levels are reduced**.**	Birsoy et al. (2013)
Apoptosis pathway	SRTFs	This protein transcribes Bcl-2 and Bcl-xL genes.	SRTF activation is inhibited.	Green and Evan, (2002)
	HDACs	Bcl-2 and Bcl-xL must be activated.	Changing the expression of HDACs.	DAMASKOS et al. (2017)
RAS pathway	Nucleotide exchange	RAS signaling activation	Nucleotide exchange is inhibited.	Huang et al. (2015)
	GRB2	RAS signaling activation	preventing the interaction of GRB2 receptors.	Ahmed et al. (2015)
	SHC	Mediate between GRB2 and the receptor.	SHC auto-phosphorylation is reduced.	Niemitz, (2013)
NF-ĸB pathway	IKK kinase complex	NF-ĸB activation requires this part.	IKK expression is reduced.	Strickson et al. (2013)
	Proteasome	This protein aids in the development of NF-ĸB.	Proteasome inhibition.	Kubiczkova et al. (2014)

## Signaling pathway

It explains various chemical reactions, such as cell division or cell death, a process in which a community of molecules in a cell collaborate to regulate a cell process. When a chemical, growth factor, or hormone, interacts with a particular protein receptor on or within the membrane, a cell gets signals from its surroundings. It activates another molecule after the first molecule on the route receives a call. Until the very last molecule is stimulated and the cell work is completed, this step is replicated through the whole signaling cascade. Abnormal signaling pathway activation can contribute to illnesses such as cancer. By targeting particular molecules involved in these pathways, drugs are being produced. Such medicines can help to prevent cancer cells from rising (National Cancer Institute, 2022).

### Notch signaling

**FIGURE 2:**
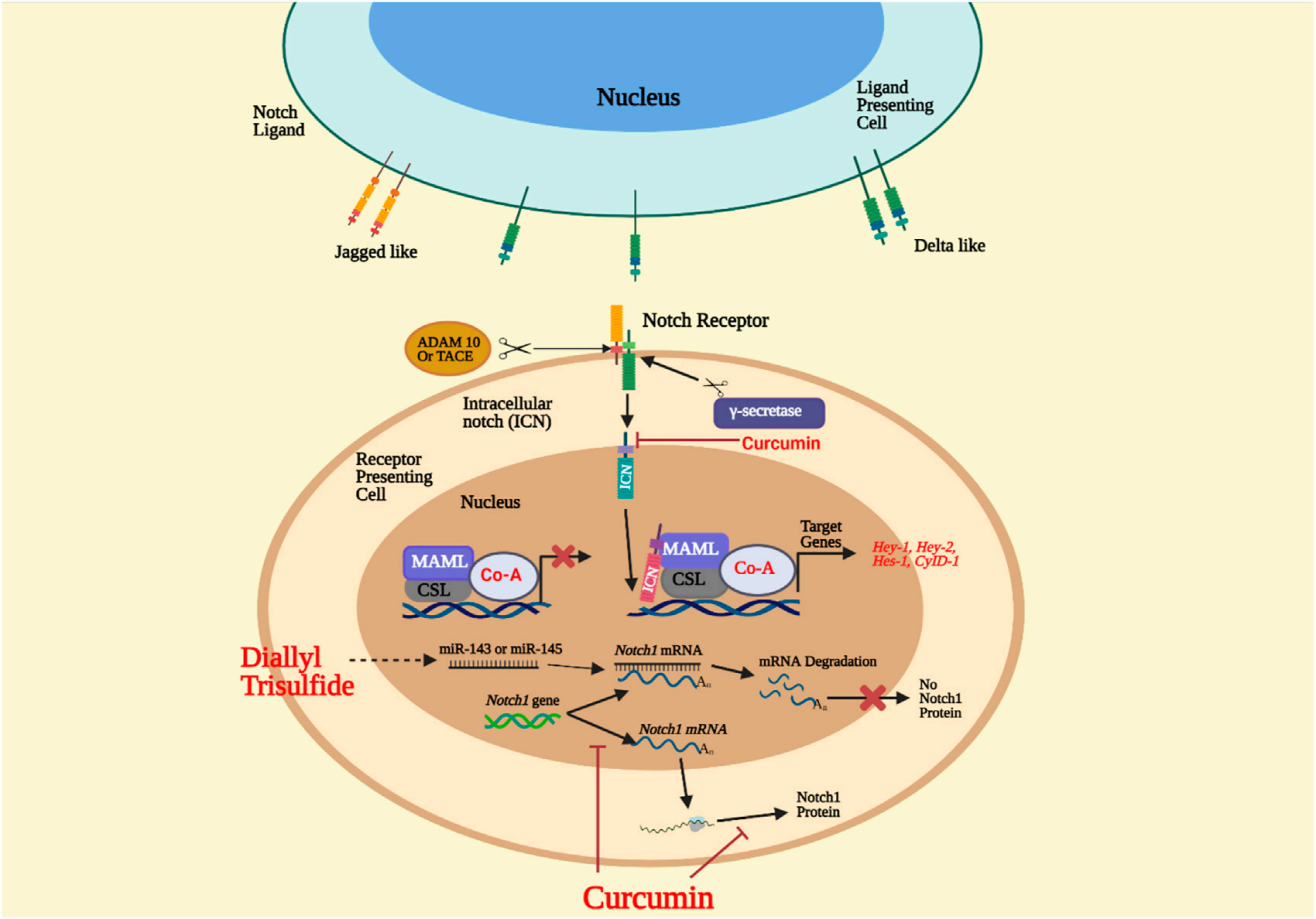
Notch signaling pathway. The ligand on the receiving cell connects the cell that acts as a presenter for the notch receptor. ADAM metalloprotease and γ-secretase split the Notch extracellular truncated (NEXT) domain, forming the notch intracellular domain (NICD). NICD is introduced into the nucleus and is a complex of transcription factors CSL 9 CBF1/hairless/lack 1 and transcriptional coactivator of mastermind-like proteins (MAML). The complex would then activate the target gene’s transcription. Treatment with diallyl trisulfide (DATS) boosts the expression of tumor suppressor microRNAs miR-143 and miR-145. When microRNAs bind to Notch1 mRNA, the mRNA is degraded, but the Notch1 protein is not translated. Curcumin inhibits Notch1 transcription and expression and the nucleus’ Hes-1, Hey-1, and Hey-2 genes (Angulo et al., 2017).

The Notch signaling pathway has been around for a long time. It has remained relatively unchanged during evolution that many organisms play a significant role in the growth of the embryo and the baby after birth. The pathway has several pleiotropic implications, affecting critical organ growth processes and adult stem cell self-renewal control, resulting in tissue homeostasis (Kopan and Ilagan, 2009). This pathway, however, is vulnerable to irregular signaling due to its multifunctional structure and has been related to a host of human diseases, including developmental syndromes and cancer. Notch signals are a well-organized, multi-tiered, closely controlled cascade of cell/tissue signaling occurrences. For its maturation, activation, and execution, it requires different components. Four receptors, known as Notch-1 to Notch-4, and five DSL (Delta/Serrate/Lag-2) ligands, known as Jagged-1 and Jagged-2 (Jag-1 and Jag-2) and Delta-like-1, Delta-like-3, and Delta-like-4, are in the Notch signaling family (Dll-1, Dll-3, and Dll-4) (Angulo et al., 2017). Transmembrane proteins, ligands, receptors, and pathway activation happens when an adjacent cell ligand communicates with the receptor (Ehebauer et al., 2006). The encounter causes conformational modifications in the ligand-receptor complex, which reveal a notch receptor site to proteolytic divide through the tumor necrosis factor-alpha converting enzyme (TACE/ADAM17/CD156q) and metalloprotease part, or ADAM (Figure 2). This cleavage results in the membrane-attached notch extracellular truncation (NEXT) fragment, a vital regulatory step in notch activation and signaling, which is located in the negative regulatory area of the notch receptor extracellular domain (Okajima and Matsuda, 2006; Sato et al., 2007).

Notch signaling is uncommon in many cancers, and tissue and cell history-based may play complex oncogenic or tumor-suppressive roles in multiple tumors (xi et al., 2020). Notch receptor mutations cause gain-of-function (for example, in malignant hematological disorders (Arruga et al., 2018; Sorrentino et al., 2019)) or loss-of-function (for example, cancer of the bladder (Goriki et al., 2018) and carcinomas of squamous cells (Zhang et al., 2016)). They are a fundamental cause of dysregulation of Notch signaling. The mechanisms that control Notch signaling by four Notch receptors, as well as their distinct functions in the progression, occurrence, and recurrence of cancer, to date have been well known, indicating that Notch receptor-based therapeutic approaches may be helpful (xi et al., 2020).

It is generally acknowledged that Notch signaling is crucial for maintaining the harmony between cell proliferation, survival, apoptosis, and diversification, which has an influence on the growth and operation of numerous organs. As a result, Notch malfunction inhibits distinction and eventually leads undifferentiated cells onto malignant transformation (Jubb et al., 2010; Wang et al., 2010; Espinoza and Miele, 2013). In fact, a number of information indicate that modifications in Notch signaling may be connected to a variety of human cancers. Furthermore, Notch receptors and ligands have been identified to serve as prognostic indicators in human cancers (Wang et al., 2009; Moretti and Brou, 2013). It is really remarkable that Notch signaling may play either an anti-proliferative or an oncogenic role in the development of tumors, depending on the situation. A limited fraction of tumor forms, including human hepatocellular carcinoma, medullary thyroid, small cell lung cancer, skin cancer, and cervical cancer, have revealed that notch signaling is anti-proliferative (Anusewicz et al., 2021; Christopoulos et al., 2021; Zhou et al., 2022).

### WNT/β-catenin signaling

Organogenesis, cell proliferation, cell fate determination, and stem cell renewal are regulated by the Wnt signaling pathway, which is strongly conserved (Angulo et al., 2017). Wnt signaling is traditionally classified as either β-catenin-dependent (canonical, Wnt/β-catenin pathway) or β-catenin-independent (noncanonical, Wnt/planar cell polarity [PCP] and calcium pathway) (Grumolato et al., 2010; Katoh, 2017). Skin, breast, and colon cancers may be caused by changes and dysregulation in the Wnt pathway (Angulo et al., 2017). Wnt signaling regulates gene transcription and cell-to-cell adhesion, and β-catenin is a critical player in this process. Degradation and phosphorylation ensure that the protein’s amount remains stable—amino acid substitutions occurring as a result of β-catenin mutations, resulting in the protein’s improper phosphorylation Table 2. The ubiquitin ligase E3 does not accept the phosphorylated protein properly. As a result of the dysregulation of the Wnt pathway, βcatenin accumulates without being destroyed and then translocates to the nucleus, stimulating oncogene transcription (Zou et al., 2015), *c-Myc, cyclin D1*, and *survivin* (an apoptosis inhibitor) are examples of expressed downstream genes. Wnt glycoproteins tie to the Frizzled receptor family of extracellular, transmembrane proteins. The signal then stimulates the cytoplasmic protein Disheveled (Dsh/DV1). Wnt then divides into three signaling pathways: canonical and noncanonical Wnt/Ca^2+^, noncanonical planar cell polarity (Komiya and Habas, 2008).

**TABLE 2:** Impact of natural compounds affecting significant stem pathways cell signaling (Angulo et al., 2017).

Major paths for signaling	Components	Objective	Effect	References
Notch	Curcumin	The notch-1 and downstream genes Hes-1, Hey-1, and Hey-2 mRNA speed downregulate transcriptions and translations.	Causes apoptosis by increasing reactive species of oxygen.	Tu et al. (2012)
	Diallyl trisulfide	Intracellular domain targets Notch-1	Decreases downstream gene expression of Notch. Enhances expression of microRNAs (miR143 and miR-145) possible tumor suppressants and reduces tumor support for miR-21 MicroRNA.	Tutelyan and Lashneva, (2013)
WNT/β-catenin	Resveratrol	histone H2AX; β-catenin	Apoptosis in ROS cells triggers telomere instability and DNA disruption by lowering -catenin mRNA and protein expression and c-Myc Histone H2AX phosphorylation.	Angulo et al. (2017)
Hedgehog	Cyclopamine	SMO-binding	Signal transduction to GLIS is prevented.	Kumar and Fuchs, (2015)
PI3/AKT	Sulforaphane	AKT and ERK	It suppresses phosphorylation of ERK and AKT and induces apoptosis by arresting cells in the G2/M process.	Aggarwal et al. (2007)

### Hedgehog signaling

**FIGURE 3:**
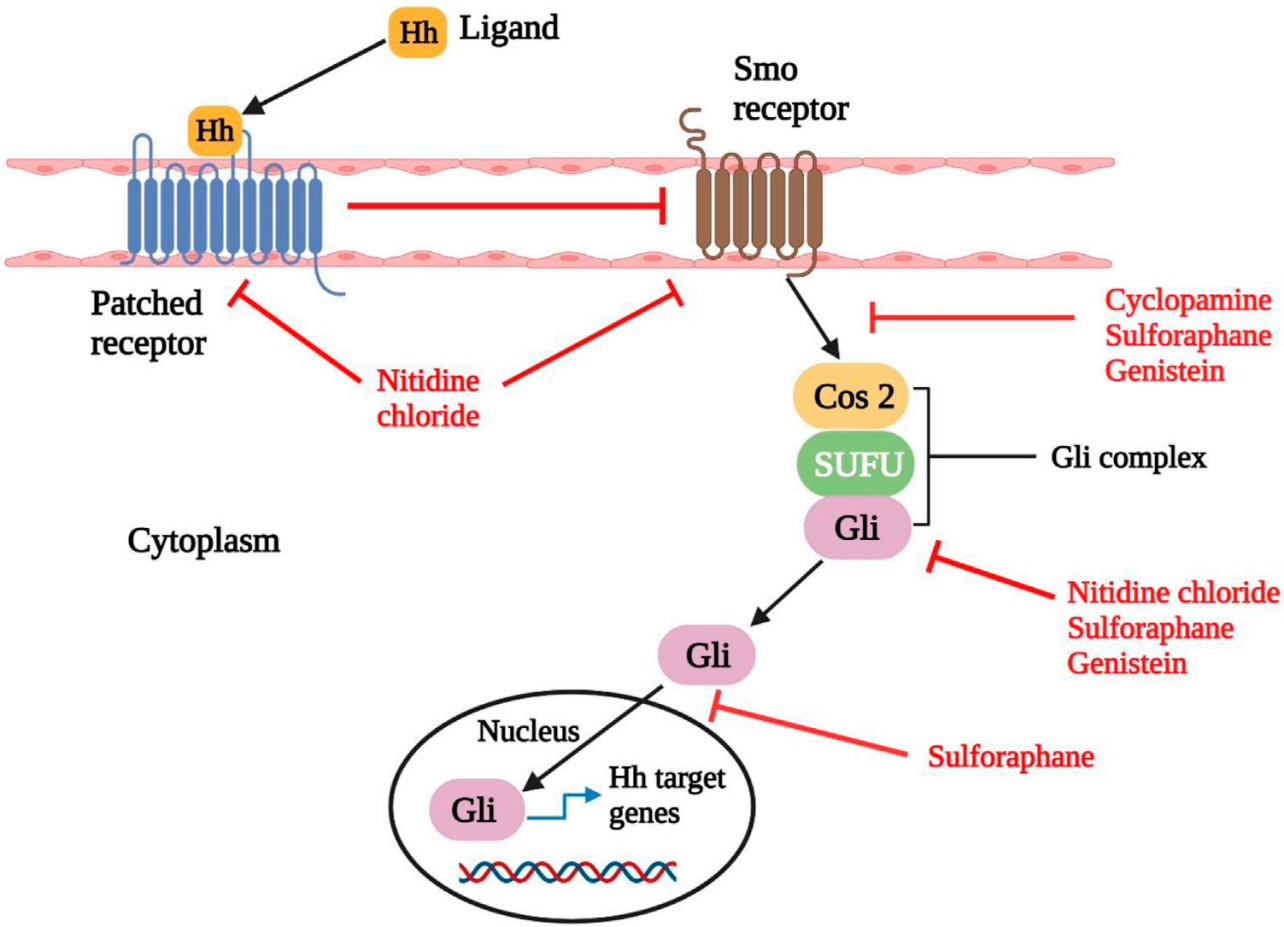
Hedgehog signaling and its interference in natural compounds. Upon binding of Hedgehog ligand (Hh) to Patched receptor, Smo is activated (Pathched generally inactivates Smo when it is not engaged with ligand). Then Smo brings about the translocation of Gli protein into the nucleus, subsequently causes the transcription of downstream genes. Compounds such as cyclopamine and genistein decrease the expression of Smo. Whereas nitidine chloride downregulates the expression of both Smo and Patched receptors. On the other hand, sulforaphane, nitidine chloride and genistein decrease the expression of Gli protein. Sulforaphane also reduces the expression of Smo, Gli and inhibits the nuclear translocation of Gli. Smo: Smoothened; Cos 2: Costal-2; SUFU: Suppressor of fused homolog; Hh: Hedgehog ligand (Skoda et al., 2018; Das et al., 2019).

The signaling pathway of Hedgehog (Hh), also called the Hedgehog-Gli (Hh-Gli), Hedgehog-Patched (Hh-Ptch), or Hedgehog-Patched-Smoothened (Hh-Ptch-Smo), is an evolutionarily conserved signaling pathway that transmits signals from the cell membrane to the nucleus (Table 2). Invertebrates and vertebrates both use the Hh signaling pathway to help them usually evolve (Varjosalo and Taipale, 2008). In the human body, the Hh pathway is inactive mainly or only moderately active. It can be triggered if necessary, such as in the cure of the wound (Le et al., 2008). It also promotes the growth of somatic stem cells and pluripotent stem cells required to reconstruct tissue such as skin, mammary, erythropoietic, neural, and pulmonary stem cells, and epithelial cells in internal organ systems. As a result, Hh signaling is needed for lung epithelial regeneration, exocrine pancreas cell regeneration, and prostate epithelial regeneration (Skoda et al., 2018). The Hh signaling pathway is only found in primary cilia (PC), which are microtubule-based organelles that protrude from the cell surface and receive chemical, mechanical, and thermal signals (Plotnikova et al., 2008). The PC contains all Hh signal transduction pathway components (Michaud and Yoder, 2006). Natural substances including Cyclopamine, Nitidine Chloride, Sulforaphane, and Genistein may be able to kill CSCs by specifically targeting the Hedgehog pathway components and inactivating the signaling cascade (Figure 3) (Cross and Bury, 2004; Varjosalo and Taipale, 2008; Rodova et al., 2012).

Hh signaling has been implicated in multiple stages of carcinogenesis in various tumors, according to several reports. The activation of the signaling pathway is seen in the early stages of pancreatic and esophageal cancers and metastatic tumors (Ma et al., 2006; Bailey et al., 2009). Regulation of the Hh signaling pathway is connected to tissue invasion and increasing metastatic risk in other cancers, such as prostate cancer and gastric cancer. Inhibition of the Hh signaling pathway inhibits tumor cell proliferation in prostate and gastric cancers, according to those studies (Karhadkar et al., 2004; Sheng et al., 2004).

### PI3K-AKT-mTOR pathway

Many cellular stimuli activate PI3K/Akt/mTOR, which controls essential cellular functions like translation, transcription, proliferation, development, and survival. Disrupted activation of the PI3K/Akt pathway has been linked to various human cancers, making it essential for producing new antitumor drugs (Porta and Figlin, 2009). Several studies have suggested that PI3K plays a part in cancer cell survival at different levels (Asati et al., 2016). The loss or downregulation of PTEN, an essential tumor suppressor protein that encodes phosphatidylinositol-3,4,5-triphosphate (PIP3) 3’-phosphatase, has been shown to stimulate the PI3K pathway (Li et al., 1997; Sansal and Sellers, 2004). Akt is phosphorylated at T308 and S473, while the binding of specific cytokines stimulates PI3K to their receptors. Phosphorylation of Akt facilitates the downregulation of some downstream substrates, such as Bad and GSK-3b, which may contribute to cancerous transformation (Chang et al., 2003). One of the essential functions of Akt during cancer cell proliferation is the aggregation of the cyclin D1 protein, which is mainly regulated by the loss of kinase activity of GSk-3b due to Akt phosphorylation (Diehl et al., 1998). The activation of the PI3K/Akt/mTOR signaling pathway has been linked to the pathogenesis of multiple cancers. It indicated that targeting individual components of this pathway, such as PI3K, phosphoinositide-dependent kinase-1 (PDK-1), Akt, and mTOR (mammalian Target of Rapamycin), may be a possible cancer therapy technique (Asati et al., 2016). According to new research, Ras appears to play an essential role in activating the PI3K/Akt pathway and controls a variety of downstream substrates (Sasaki and Firtel, 2005).

### Ras-Raf-MEK-ERK pathways

**FIGURE 4:**
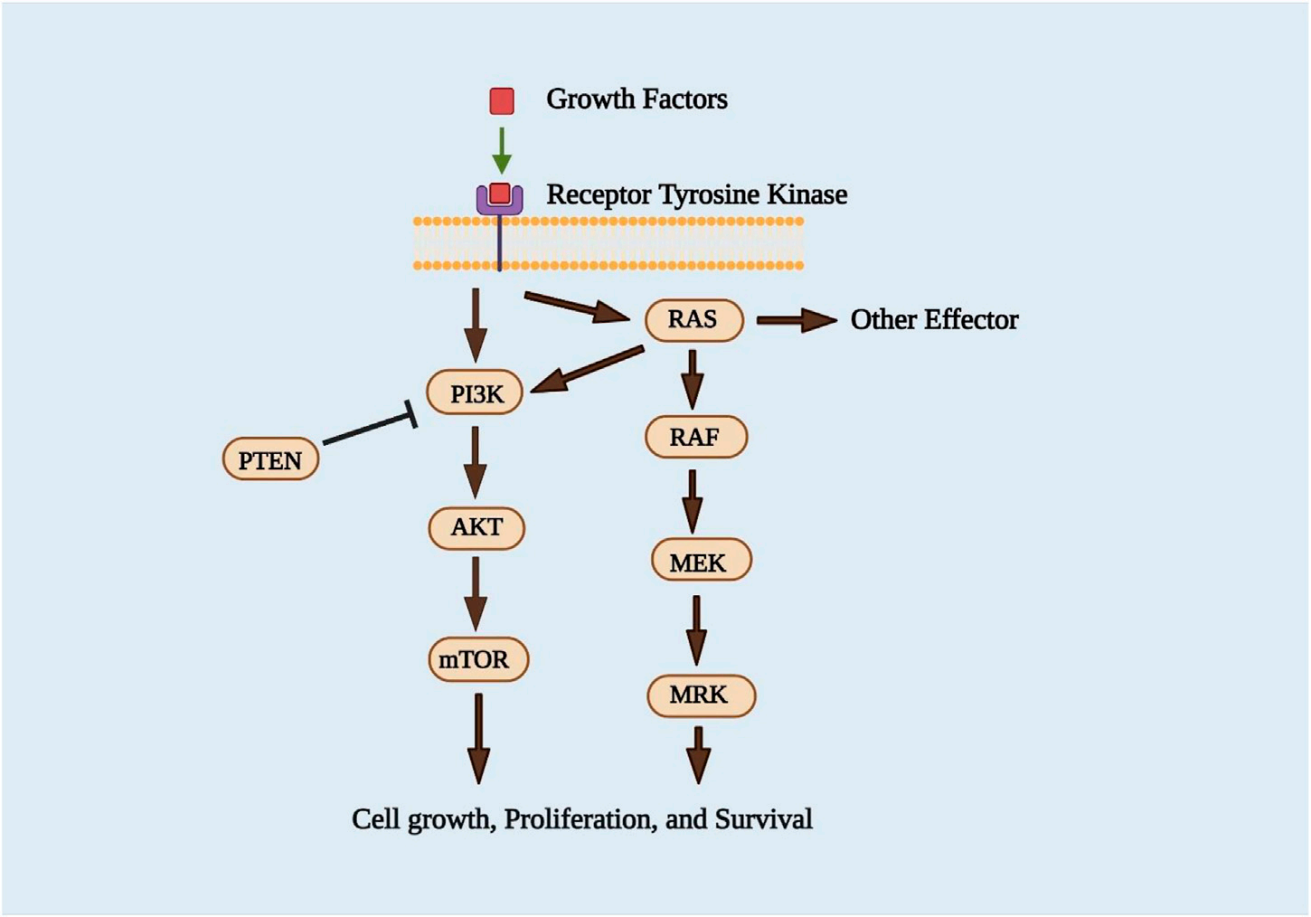
PI3K/Akt/mTOR and Ras/Raf/MEK/ERK signaling pathways (Asati et al., 2016).

The Ras/Raf/MEK/ERK pathway is also essential for cell survival at various stages of cancer. In around 30 percent of human cancer, Ras system mutations lead to the expression of Ras proteins that are constitutively active (Stirewalt et al., 2001). Upstream activation by the epidermal growth factor receptor (EGFR) and Ras small guanosine triphosphatases promotes cell proliferation, survival, and metastasis (GTPases) (Roberts and Der, 2007). Phosphorylation positively regulates the activities of ERK 1/2, which include Serine/Threonine Kinases, which MEK1 and MEK2 mediate (Chang et al., 2003). The Ras/Raf/MEK/ERK signaling pathway’s phosphorylated ERK (pERK) is a well-known downstream portion. It translocates to the nucleus after being phosphorylated, causing changes in gene expression and regulating various transcription factors such as the Ets family transcription factors (Elk-1) (Roberts and Der, 2007). The Ras/Raf/MEK/ERK signaling cascades are also crucial in regulating gene expression and preventing apoptosis by transmitting signals from growth factor receptors (McCubrey et al., 2007). Since tumors frequently have abnormal expression, they may be candidates for slight molecule inhibition (Ripple et al., 2013). In recent years, two critical methods for identifying Ras inhibitors have been systematically pursued. Various inhibitors for Ras downstream effector signaling have been developed in the first approach, with hard work focusing on the ERK/MAPK pathway. The second strategy aimed to prevent Ras membrane interaction by blocking post-translational modifications (Roberts and Der, 2007). The development of inhibitors of the PI3K/AKT/mTOR and Ras/MEK/ERK pathways (Figure 4), which are known to be the critical transducers of oncogenic signals in tumor progression, has been crucial (Jokinen et al., 2012).

## Role of natural compounds in cancer prevention

Plants provide a vast pool of natural ingredients, displaying significant structural diversity, and offer a wide range of new and exciting chemical species, as well as a long tradition of use in the treatment of several diseases (Table 3). According to a survey, 80% of the world’s population already uses plant-derived drugs to meet their healthcare needs (Fulda, 2010). It is also been recorded that natural products, their derivatives, or analogs account for 50% of all medications in clinical use, with plant-derived active ingredients accounting for 74% of the most effective drugs (Mandal and Jaganathan, 2009; Hsu et al., 2010). In modern medicine, more than 3,000 plant species have been long-established to be used to treat cancer (Millimouno et al., 2014). Taxol, irinotecan, vincristine, vinblastine, eribulin, topotecan, trabectedin, and cytarabine are a few examples (Atanasov et al., 2015; ÁK, 2016). The prevention and treatment of cancer with one or more natural substances continue to attract significant interest. Combination treatment entails the use of two or more agents at the same time. Because of the disease’s many targets nature, monotherapy has poor success in treating most adult malignancies. It has limited value in prevention due to the diverse paths through which cancer might arise (AS and WF, 1975). Tissue toxicity frequently restricts the usage of a single agent. Other than dosage, natural substances are more likely than pharmacologic treatments to be connected with hazardous consequences, such as adulteration, substitution, contamination, and lack of standardization (JH, 1990). Multiple chemotherapeutic drugs and, more recently, chemotherapy with targeted biologic therapy have been used in cancer treatment as combinations (Bartlett and Chu, 2012).

**TABLE 3:** Mode of action and chemical structure of natural compounds.

Natural compounds	Chemical structure	Mode of action	Cancer Lines	References
Curcumin	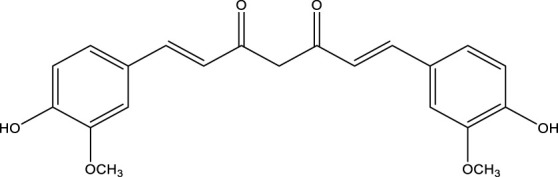	Stopping tumor cell invasion and proliferation by inhibiting a number of biological signaling pathways, hence inducing apoptosis.	Colorectal, breast, pancreatic, prostate, brain cancer	Kunnumakkara et al. (2017); Tomeh et al. (2019)
Diallyl trisulfide	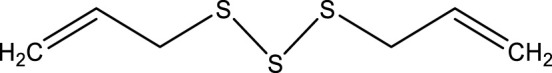	Controls a number of processes that are characteristic of cancer, including the cell cycle, apoptosis, angiogenesis, invasion, and metastasis. Arrest cancer cells at several cell cycle stages, with the G2/M arrest receiving the most attention.	Colorectal, lung, myeloma, prostate cancer	Puccinelli and Stan. (2017a); Bansal et al. (2018)
Resveratrol	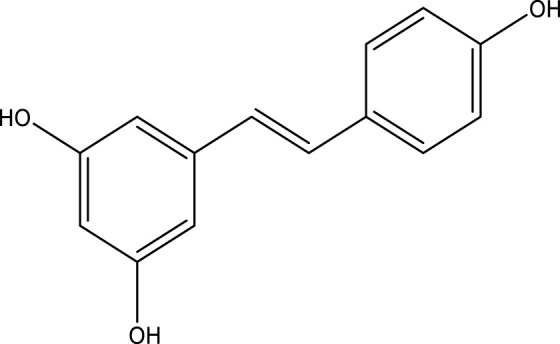	Since it blocks the monooxygenase cytochrome P450 isoenzyme CYP1 A1, the enzyme responsible for the liver’s metabolism of xenobiotics, it acts as a blocking agent by preventing the development of procarcinogen into carcinogen.	Lymphoid, cervix, breast, skin, stomach, prostate, colon, pancreas, thyroid carcinoma cell cancer	Chun et al. (1999); Diaz-Gerevini et al. (2016); Varoni et al. (2016); Ko et al. (2017)
Apigenin	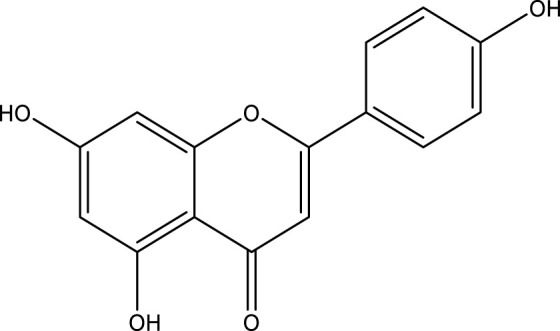	Activate cell apoptosis and autophagy, cause cell cycle arrest, inhibit cell migration and invasion, and stimulate an immunological response to control a variety of human malignancies both *in vitro* and *in vivo*.	Colorectal, lung, prostate, breast, ovarian, melanoma, Glioblastoma, Pancreatic, cervical cancer	Yan et al. (2017a)
Cyclopamine	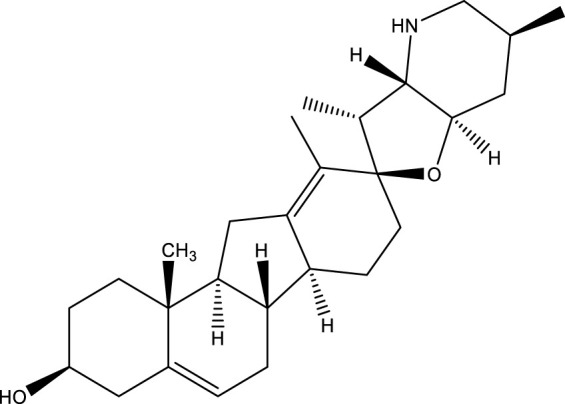	Bloked the hedgehog signaling pathway (Hh).	Prostate, pancreas, breast cancer	Chai et al. (2013; Aphios (2022)
Genistein	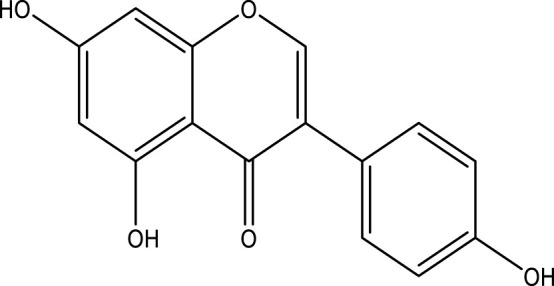	Activates the transcription factor CCAAT/enhancer-binding protein homologous protein (CHOP) to cause apoptosis by increasing the production of the glucose-regulated protein 78 (GRP78), which in turn boosts the activity of protein kinase R-like ER kinase (PERK).	Breast, lung, and colon cancer	Tuli et al. (2019)
Quercetin	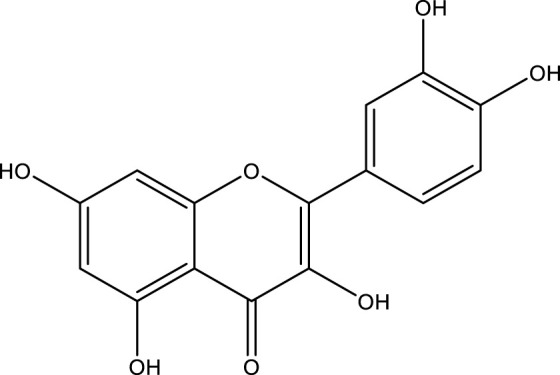	Caspase activation and apoptotic cell death are the end results of activating p53, which causes the overexpression of Bax and the downregulation of Bcl-2 in tumor cells. Quercetin alters mesangial cells’ apoptosis by preventing the activation of the JNK and other ERK pathways.	Breast, colorectal, stomach, head, melanoma, ovarian, lung, leukemia and neck cancer	Khan et al., (2016); Hashemzaei et al. (2017)
Tetrandrine	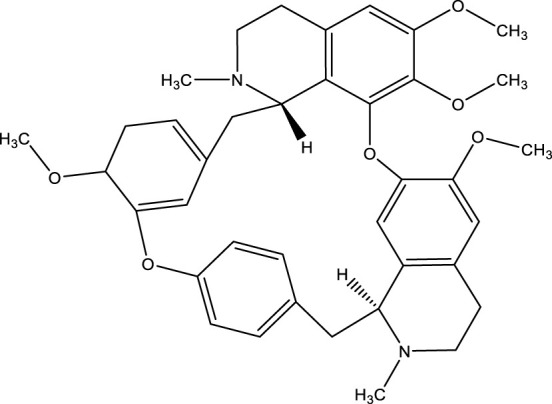	Induce apoptosis in a dose-dependent manner leading cancer prevention.	Breast, liver, leukemia, colon, pancreatic cancer	Shang et al. (2021); Aphios (2022)

Although a single chemical extracted from nature may not be ideally efficient in preventing or treating cancer, combination therapy utilizing lower dosages with no or decreased toxicity may be beneficial (Bode and Dong, 2009). If possible, use a combination of medicines that targets distinct disease pathways and mechanisms. When possible, combination therapy should use medications that do not cross-resistance and have overlapping side effects. When feasible, tailored treatment should be utilized to match specific molecular changes (Nikanjam et al., 2017). Maximizing response rates while minimizing standard tissue damage is achieved by targeting several routes or the same pathway through a different method (Gravitz, 2011). Tumor resistance can be overcome better with combination therapy than with monotherapy, as most adult malignancies display substantial genetic variability (Piccart, 2015).

## Chemo preventive agents and anticancer compounds

Chemoprevention has become a successful and proactive medical approach to minimizing cancer incidence as a method of cancer control. The development of the disease may be avoided entirely or slowed down by using a nontoxic naturally derived item (Amin et al., 2009). Cancer therapy with economically obtainable manufactured chemotherapeutic agents is restricted due to extreme side effects (Surh and Chun, 2007). Phytochemicals obtained from some kind of food items which are known as chemopreventive agents such as Curcumin, Diallyl trisulfide, Resveratrol, Apigenin, Cyclopamine, Genistein, Quercetin, Tetrandrine, Silibinin, Thymoquinone. When opposed to their synthetic counterparts, natural goods are typically thought to be harmless and free from serious adverse consequences at therapeutic doses, which drives up demand for them dramatically (Pradhan et al., 2020). Phytochemicals obtained from natural sources could be a mix of other compounds. This mixture of other compounds is hard to separate or isolate from target compound. These may interfere with the effectivity of the target compound. The technology of isolating and purifying bioactive or target compounds from plants has recently undergone new advancement. This cutting-edge method allows for a parallel between the creation and accessibility of numerous sophisticated bioassays on the one hand, and the provision of exact techniques for isolation, separation, and purification on the other. Finding a suitable approach that can screen the source material for bioactivity, such as antioxidant, antibacterial, or cytotoxicity, while combining simplicity, specificity, and speed is the aim while looking for bioactive or target compounds (Altemimi et al., 2017). It has been shown that these agents prevent the propagation of carcinogenesis, inhibit the signaling pathways of the development factor, trigger apoptosis, inhibit the activation of NF-kB, AP-1, and JAK/STAT pathways, inhibit angiogenesis, and inhibit cyclooxygenase-2 **(** Table 3 **)** (Dorai and Aggarwal, 2004).

### Curcumin

It is also known as Diferuloylmethane. It is a light-yellow dye that is part of the polyphenol class. It is obtained from the turmeric rhizomes (*Curcuma longa* L.) (Plants of the World Online KS, 2022). It is a type of condiment used chiefly for food conservation and well-being in Asian countries. Additionally, utilized as a beauty product and in some pharmaceutical preparations (Rajasekaran, 2011). The compound had been extracted for the first time two centuries earlier, and it was used to treat many systemic conditions such as respiratory, dermatological, and gastrointestinal disorders. Because of its wide variety of functional attributes, Curcumin has been capable of performing many of these activities,“including antioxidant, antiviral, antibacterial, antifungal, anti-inflammatory, and anticancer” characteristics (Aggarwal et al., 2007).

Curcumin is likely to have a suppressing impact on cancer development by many processes including inhibiting carcinogenic activation and cancer-causing agent detoxification, preventing oxidative DNA damage, and reducing inflammation (Surh and Chun, 2007). Curcumin has been proven beneficial in all three stages of carcinogenesis (initiation, promotion, and progression). Plenty of its potential impact is obtained because of inhibiting transcription factor NF- κB and subsequent suppression of the pro-inflammatory pathways (Thangapazham et al., 2006). It has the good prophylactic and curative ability for many cancers, “including cancer of leukemia, cancer of the gastrointestinal tract, lymphoma cancer, head and neck cancer, lung cancer, genitourinary cancer, breast cancer, ovarian cancer, and skin cancer” (Anand et al., 2008).

Curcumin restrained development and facilitated autophagy and apoptosis (Fu et al., 2018). Mechanistic research has shown that Curcumin can enhance p53 and p21, thereby enabling the signaling pathway for p53. Curcumin has also been shown to denature the PI3K signaling pathway. These findings established Curcumin’s role in inhibiting GC progression, suggesting that it could be used as a GC treatment. By inhibiting the expression of MMP-2/9/9, Curcumin has regulated the invasiveness of oral squamous cell carcinoma (Lee et al., 2015). It also prevented the EMT process by overexpressing p53 function in oral squamous cell carcinoma (OSCC) cells. Curcumin could be used for OSCC medicinal services as an adjunctive method (Li et al., 2015). A higher therapeutic technique for treating human cervical cancer could be Curcumin combined with paclitaxel l (Wang et al., 2019).

### Diallyl trisulfide

Food items including fruits, vegetables, and condiments have been used to both prevent and treat a variety of ailments, such as infections, irritability, and wounds. In recent times, it has been shown that bioactive compounds obtained from all of these foods have anti-microbial, anti-inflammatory, and anticancer effects. Plant species of the *Allium* genus, like garlic and onions, are renowned for their therapeutic quality for a long time (Block, 1985; Petrovska and Cekovska, 2010). Studies have found that organosulfur compounds (OSCs) are the principal bioactive agents responsible for positive effects. Diallyl trisulfide (DATS), a bioactive OSC contained in garlic, can be used to attenuate disease conditions like cancer, infection, and metabolic processes (Mikaili et al., 2013). Cell-cycle deregulation mechanisms are an initiatory cancer production event that allows the unrestrained progression of the cell cycle and rapid growth of the tumor. Few numbers of researching have shown that DATS mediates G2/M-stage cell cycle arrest. DATS induced cell cycle arrest by increasing reactive oxygen species (ROS) (Hosono et al., 2005; Xiao et al., 2005; Antosiewicz et al., 2006). The DATS increase apoptosis which is an effective anti-cancer treatment intended to slow tumor growth. Evidence is mounting that DATS causes cancer cells to respond to cell death signals like oxidative stress, DNA damage, and cellular damage. Results across a variety of cancer cell types have shown that DATS therapy activates the intrinsic apoptotic pathway. Numerous studies have demonstrated increased activation of the apoptosis-regulating protein c-Jun N-terminal kinase (JNK) following DATS incubation. Recent investigations have shown that DATS therapy can inhibit the proteins related to invasion and migration. Following DATS treatment, levels of Tissue Inhibitors of Metalloproteinases (TIMP) -1 and -2, known Metrix Metalloproteinases (MMPs) inhibitors, increased, resulting in improved tight connection formation between bladder cancer cells. The progression of breast and prostate cancers is known to be significantly influenced by estrogen and androgen hormone signaling, respectively. Estrogen sensitivity and HER-2 expression are significant determinants of patient outcome in breast cancer. Studies with breast cancer cell lines that varied in their sensitivity to estrogen and HER-2 status revealed reduced cell viability after DATS therapy (Puccinelli and Stan, 2017b).

### Resveratrol

It is also named as 3,5,4′-trihydroxytrans-stilbene. Resveratrol is a naturally appearing phytoalexin and phenol derived from various plants in response to wounds and microbes. Other sources like mulberry, raspberry, blueberry, raisin, and peanut (Ferrucci et al., 2016). Initially, it was separated from the roots of the white hellebore. This phenol is a practical component of the root from *Polygonum cuspidatum* (Dandawate et al., 2013). Resveratrol has antioxidant, anti-inflammatory, and antiproliferative effects on many cancer cells. It efficiently breaks down the enzyme and non-enzyme-generated superoxide, hydroxyl, and peroxynitrite fundamentals and provides defense against DNA damage caused by these ROS (Leonard et al., 2003; Lee and Lee, 2006; Gagliano et al., 2010).

Resveratrol, relying on its striking restrictive impact on cellular events linked to cancer initiation, promotion, and progression, is a viable contender for cancer chemoprevention (Zhu, 2011). It promotes antitumor activity by controlling multiple cell-signaling molecules, including drug therapy transporters, proteins for cell survival, cell proliferative proteins, and signals to members of the NF-κB and STAT3 (Gupta et al., 2011). In order to effectively lead the multiple cycles of cancer from start and promotion to progression, resveratrol alters the several signal-transduction pathways that control cell growth and division, inflammation, metastasis, apoptosis, and angiogenesis. It has been discovered that resveratrol can stop processes connected to the development of cancers. For instance, resveratrol therapy reduced the production of free radicals in human leukemia HL-60 cells that were generated by 12-O-tetradecanoylphorbol-13-acetate (TPA) (Sharma et al., 1994). Studies conducted *in vitro* have demonstrated that resveratrol has an anti-proliferative impact through inducing apoptosis. Resveratrol affects the proportions of cyclins and cyclin-dependent kinases (CDKs), which inhibits the cell cycle at the G0/G1 phase. For instance, a connection has been discovered between cell cycle arrest in the G0/G1 phase inside several cancer cells and resveratrol’s suppression of cyclin D1/CDK4 (Gatouillat et al., 2010). Additionally, it has been demonstrated that resveratrol raises cyclin A and cyclin E levels, causing cell cycle arrest in the G2/M and S phases (Filippi-Chiela et al., 2011). Similar research has shown that resveratrol stops cell cycles and activates a mechanism that is dependent on p53 (Hsieh et al., 2011). Tumor metastasis is caused by a number of processes that are involved in the progression of the tumor. The deletion or mutation of many genes promotes the growth of malignant cancers. Proteolytic enzymes like matrix metalloproteinases are used by cancer cells to invade and spread by destroying the extracellular matrix (ECM) and basement membrane (MMPs). MMP-2 and MMP-9 are two of these enzymes that are overexpressed and regulate cell invasion and metastasis in a range of malignant cancers (Nelson et al., 2000). Resveratrol may decrease the expression of angiogenesis indicators like VEGF, EGFR, and FGF-2 as well as MMPs, particularly MMP-9 (Trapp et al., 2010; Ko et al., 2017).

### Apigenin

It is also known as 4′,5,7-trihydroxyflavone. Apigenin is a naturally appearing glycoside that belongs to the class of flavone. Various vegetables and fruits produce the compound, including onions, tea, wheat sprouts, and oranges (Tutelyan and Lashneva, 2013). It has characteristics as being anti-oxidant, anti-inflammatory, anti-growth, anti-mutagenic, anti-carcinogenic, and anti-progression (Patel et al., 2007). Previous researches have shown that apigenin prevents tumor proliferation, invasion, and tumor development in prostate cancer cells (Shukla et al., 2015). Dimethyl benzanthracene-induced skin tumors are suppressed by topical use of apigenin (Wei et al., 1990). It also decreased the prevalence of UV-induced cancer and improved tumor-free survival studies (Birt et al., 1997). Apigenin encourages metal chelation, searches for free radicals, and boosts phase II detoxifying enzymes in cell culture and *in vivo* malignant tumors (Elliott et al., 2000). In the PC-3 tumor model, therapy with apigenin ended in 32 percent and 51 percent suppression of tumor growth (Wei et al., 1990). Studies conducted *in vitro* have demonstrated that resveratrol has an anti-proliferative impact through inducing apoptosis (Liu et al., 2015). Apigenin’s anti-cancer properties and minimal toxicity have recently attracted a lot of attention. Apigenin has been shown to inhibit a number of human malignancies both *in vitro* and *in vivo* by a variety of biological mechanisms, including inducing cell apoptosis and autophagy, cell cycle arrest, inhibiting cell migration and invasion, and eliciting an immunological response (Yan et al., 2017b).

### Cyclopamine

Cyclopamine is a steroidal alkaloid that can counteract cancers including prostate, gastrointestinal, breast, and osteosarcoma cancer (Qualtrough et al., 2004; Warzecha et al., 2012; Lü et al., 2014; Zhu et al., 2015). It was separated from the plant corn lily *(Veratrum californicum*) in the late ‘60s (Lee et al., 2014). Cyclopamine binds to the receptor smoothly, inhibiting additional signal transduction to the GLI5 destination gene (Warzecha et al., 2012). Apart from having a significant role in the development of an embryo, improper initiation of the sonic hedgehog signaling pathway in different cells has been related to nevoid basal cell cancers and many cancers, including basal cell cancers medulloblastoma, and rhabdomyosarcoma (Chen et al., 2002; Heretsch et al., 2010). Nevoid basal cell cancer has been identified as a hereditary condition with several basal cell carcinomas, malignant and benign tumors, and malformations (Bale and Yu, 2001). In a way, the Shh (Sonic hedgehog) signaling pathway’s disruption could effectively cure cancer because it can prevent the development of tumors without some typical side effects of conventional carcinogenic therapy. It has been demonstrated that cyclopamine can prevent tumor development. Numerous cancers, including prostate cancer, pancreatic cancer, ovarian cancer, gastrointestinal cancer, lung cancer, and basal cell carcinoma, are linked to aberrant Hedgehog (Hh) signaling. One of the most actively researched targets for cancer therapy is the Hh signaling system, and several drugs that block Hh signaling are currently undergoing clinical trials to treat a variety of malignancies. More people die from lung cancer than from the next three most frequent malignancies combined (colon, breast, and prostate). Understanding the role of Hh signaling in development and cancer has benefited greatly from the discovery of the first drug to suppress Hh signaling, cyclopamine (Alam et al., 2016).

### Genistein

Also known as 4′,5,7-Trihydroxyisoflavone. It is a phytoestrogen in soybeans and tofu, soy milk, and soy sauce and is an effective therapeutic agent for cancer (Fotsis et al., 1995; Li et al., 2010). Genistein therapy was found to prevent the development of several cancerous cells by enhancing apoptosis, triggering delayed cell cycles, and regulating intracellular signaling pathways (Li et al., 2010). The potency of intakes of genistein for breast, prostate, and colorectal cancers has been shown in epidemiological reports (Hwang et al., 2009). Modern research has shown that genistein disrupted EGF-induced proliferation by modulation of the PI3K/Akt routes in cancerous colon cells (Qi et al., 2011). Genistein is attributed to cell cycle blockage and cell death in breast cancer cell lines (Magee and Rowland, 2004). It can induce the death of breast cancer cells through the accumulation of intrinsic copper ions and reactive oxygen species (ROS) (Ullah et al., 2011). Genistein is a potent anti-angiogenic compound that can suppress VEGF-induced endothelial cell activation by reducing the function of protein tyrosine kinase (PTK) and the activation of MAPK (Yu et al., 2012). Anti-proliferative activities of genistein obtain from reduced insulin-like growth factor receptor (IGFR) phosphorylation and IGF signaling, which suppresses cell development (Lee et al., 2012). When genistein is used in combination with chemotherapeutic substances, “including letrozole, resveratrol, vitamin-D, paclitaxel, erlotinib, doxorubicin, and cetuximab,”, it may be of greatest benefit (Park et al., 2010). Genistein induces ER stress by upregulating the expression of the protein glucose-regulated protein 78 (GRP78). The transcription factor CCAAT/enhancer-binding protein homologous protein (CHOP), which induces apoptosis, is then triggered by the activation of PERK (Tuli et al., 2019). By influencing the nuclear translocation of phosphorylated ERK molecules, genistein reduced the activity and proliferation of cells. ERK regulates cell growth, differentiation, and proliferation, whereas p38 is more closely associated with stress and inflammatory responses (Irrera et al., 2017).

### Quercetin

**FIGURE 5:**
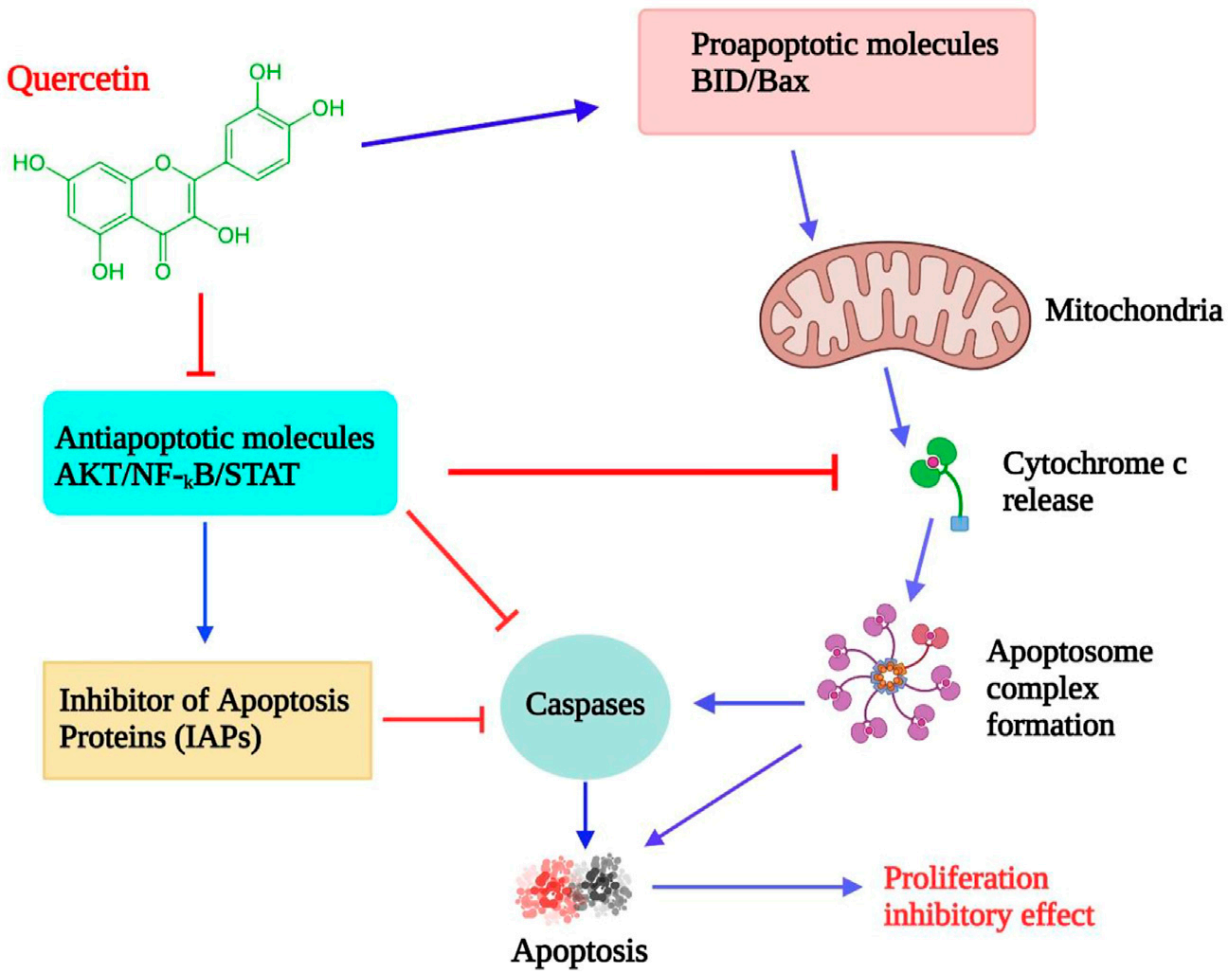
Effects of quercetin on lymphoma cells: proposed model. By inhibiting antiapoptotic signaling molecules and inducing proapoptotic proteins, which activate mitochondrial-mediated caspase activation and apoptosis, quercetin reduces cell proliferation (GULATI et al., 2006; Srivastava et al., 2016).

Quercetin is a naturally occurring bioflavonoid found in a wide assortment of foods, along with apples, grapes, berries, broccoli, onions, tea, tomatoes, nuts, barks, and leaves (Kelly, 2011). As a lipophilic substance, quercetin can pass through cell membranes and start a number of intracellular signaling pathways. The ability of quercetin to serve as both an antioxidant and a peroxidant is one of its distinctive properties (Wätjen et al., 2005). It appears to happen as O-glycosides, and the most common sugar residue is D-glucose. About 170 glycosides of quercetin were identified (Yang et al., 2001). It has several medicinal properties, “including anti-oxidant, anti-bacterial, anti-inflammatory, and anticancer” (Rafiq et al., 2015). It is an effective agent of chemoprevention due to its cardinal effect on the distinctive characteristics of carcinoma and its impact on the signaling pathways associated with tumors (Russo et al., 2012). It exhibits various activities, including modulation of the cell cycle, interacting with type II estrogen receptors, and inhibiting tyrosine kinase (Lamson and Brignall, 2000). When quercetin is administrated intravenously, It blocked lymphocyte tyrosine kinase in people with cancer and is the first inhibitory agent of tyrosine kinase tested in a clinical phase I trial (Ferry et al., 1996). It appears that quercetin may have anti-proliferative, anti-tumor, and apoptosis-inducing properties (Figure 5) (Wätjen et al., 2005; Ezzati et al., 2020).

Excessive reactive oxygen species (ROS) generated oxidative strain carries a significant factor in cancer growth. ROS serves itself as a “redox messenger” to promote the growing phases of cells at lower physiological volumes. Residual ROS could damage DNA under oxidative strain. Quercetin can prevent ROS-mediated hepatocarcinogenesis by increased activation of enzymatic and non-enzymatic anti-oxidant defense mechanisms. So, like an antioxidant, quercetin can scour ROS and lower the threat of DNA disruption and cancer development. Due to its overall antioxidant Capacity and higher reducing ability, quercetin inhibits ROS production and active nitrogen molecules in severe monocytic leukemia cells (Waris and Ahsan, 2006; Vásquez-Garzón et al., 2009; Circu, 2010; Zhang et al., 2011). In chemotherapeutic procedures, apoptosis plays an important role. Quercetin can excite apoptosis pathways both intrinsically and extrinsically (Chien et al., 2009).

### Tetrandrine

It is a bis-benzylisoquinoline (BBI) alkaloid, separated from the root of *Stephania tetrandra* (Yang et al., 2021). Tetrandrine has different beneficial effects such anti-rheumatic, anti-inflammatory, immunomodulatory, and anti-hypertensive (Yu-Jen, 2002). Collected data shows that tetrandrine has a resistant effect on several cancers, including leukemia, primary hepatic cancer, colorectal cancer, lung cancer, glioblastoma, and nasopharyngeal cancer (Lai et al., 1998; Lee et al., 2002; Kuo and Lin, 2003; Meng et al., 2004; Ng et al., 2006; Sun et al., 2007; Chen et al., 2009; Cho et al., 2009; Wu et al., 2010). Remedial actions of tetrandrine on many cancerous cells have been documented, including inhibition of growth, anti-inflammatory, modulation of the cell cycle, apoptosis stimulation, angiogenesis prevention, and the abstraction of multidrug sensitivity (Yu-Jen, 2002). Pharmacologically, a few numbers of signaling pathways or essential conditions for example, “mitogen-activated protein kinases (MAPKs), Wnt/β-catenin, PI3K/Akt, and p53,” have been documented to participate in the cancer prevention process by tetrandrine (Meng et al., 2004; NOMURA et al., 2007; Wu et al., 2010). By influencing Wnt/β-Catenin, Tetrandrine could prevent the development of the human colorectal cancer cells (He et al., 2011). Tetrandrine has also been seen to incite cytotoxicity and apoptosis of human nasopharyngeal cancerous cells through stimulating ROS-mediated mitochondrial and endoplasmic reticulum (ER) stress pathways and Akt/FOXO3-activation (Chaudhary and Vishwanatha, 2014; Zhang et al., 2015; Lin et al., 2016). Tetrandrine suppressed endometrial cancer cells in a time- and dose-dependent way, the research showed. Tetrandrine caused endometrial cancer cells to undergo apoptosis in a dose-dependent manner, according to flow cytometric analyses (Shang et al., 2021).

## Targeting kinase activity by instinctive agents in chemoprevention

Natural substances obtained from several resources can help to activate a range of biological pathways that can protect from cancer (Balunas and Kinghorn, 2005; Cragg and Newman, 2005). The catalyzation of protein phosphorylation by protein kinases is essential for controlling cellular activity. Protein kinases are manifested in different cellular components such as on the cell’s exterior, cytoplasm (e.g. Cyclin-based protein kinases), and intracellular regions (e.g. nucleus). Undesirable protein phosphorylation is involved in various disease pathways, including cancer. The construction of multi-targeted and much more efficient PTK blockers gives potential prospects for cancer treatment (Marston, 2011). So, we focused on the evaluation of two agents which are used as significant substances to prevent tumor development/Epstein Barr virus-related cancer.

### Grifolin, a potential agent for kinase inhibitor, controls cancer development by targeting ERK1/2 and DAPK1

Grifolin, an active farnesyl phenolic agent, is a secondary plant metabolite derived from the mushroom *Albatrellus confluens*. This also derives from the eatable *Boletus pseudocalopus* mushroom (Song et al., 2009). It has been proven to trigger apoptosis or cell cycle arrest in several tumor cell lines by targeting ERK or upregulation of DAPK1 via p53 (Ye et al., 2007; Luo et al., 2011). The ERK-1/2 pathway plays some crucial roles in modulating many biological functions, including cell proliferation, division, cell cycle transformation, and longevity. The majority of evidence indicates that ERK1/2 pathway activity leads to tumorigenesis or cancer development and increases cell death (Ishikawa and Kitamura, 1999). ERK modulates several transcription factors such as Elk1, c-J un, c-Myc, and c-F os. These variables restrict genetic expression, which is essential for cell cycle propagation, specifically cyclin D1 and p21 (MacCorkle and Tan, 2005). Analyzing the impacts of grifolin on the function of G1-related protein shows that the down-regulation of cyclin D1, CDK4, cyclin E, and the phosphorylation of grifolin-inducing pRB are correlated with the cell cycle blockage. Grifolin resists the multiplication of CNE1 cells by the G1 phase blockage, mediated by G1-protein regulation. Grifolin mainly has an impact on CNE1 cells involving the ERK1/2 route. At high enough doses, the ERK1/2 route could be inhibited (Ye et al., 2005). DAPK1 is a positive moderator which is apoptotic-positive (Pelled et al., 2002). It operates as a tumor suppressor because of its capability to sensitize cells to multiple apoptotic signals, “including those produced by mortal receptors, cytokines, matrix removal, and oncogenic hyperproliferation,” which are envisaged as a cell occurs tumorigenesis. DAPK1 mRNA and protein function has been up-regulated with grifolin in NPC cells in a dose-dependent way. Up-regulation of DAPK1 by grifolin can be an essential process to cause an apoptotic response in the tumor cells. Grifolin can retrieve the pro-apoptotic role of DAPK1 through the p53 route because of the higher occurrence of DAPK1 activity loss in various tumor forms (Luo et al., 2015).

### Neoalbaconol, a potent antagonist of PDK1, causes many cell deaths through the PI3K/AKT pathway

It is a new tiny-molecular compound derived from mushroom *Albatrellus confluens*, which can aim at 3-phosphoinositide-dependent protein kinase 1 (PDK1) and suppress its phosphoinositide-3 kinase (PI3-K)/Akt-hexokinase 2 (HK2) routes, which contributed to energy abolition. By locating PDK1, Neoalbaconol decreases glucose and ATP production, triggered autophagy, and induced apoptotic and necroptotic death of cancerous cells by a separate route. The probability that increased activity of Akt counteracts the energy shortage caused by Neoalbaconol, confirms the significance of the PDK1-Akt energy route in NA-induced cell death (Deng et al., 2013). The metabolic energy reconfiguration of cancerous cells is among the essential aspects. The oncogenic PI3K/AKT/mTOR route plays a significant role in reconfiguring metabolic processes in cancerous cells. PDK1 is an Akt/mTOR signaling controller, that stimulates some protein kinases belonging to the AGC kinase group, such as A, G, and C (Toker and Newton, 2000; Vivanco and Sawyers, 2002). PDK1 phosphorylates function at Ser308 in response to different cell stimulation, contributing to the Akt activation and controlling energy synthesis, cellular proliferation, cell cycle progress, and migratory. Depending on essential activities of PDK1 in tumor cells, analysts lately demonstrated that PDK1 acts as an appropriate therapeutic tool for anticancer and established multiple PDK1 blockers, including AR-12 and GSK233447, for the killing of cancerous cells (Peifer and Alessi, 2008).

So, restructuring cellular energy metabolic processes by locating the PDK1/PI3K/Akt signal pathway, NA is concerned with NA-mediated apoptotic and necroptotic cell destruction. These observations demonstrate that NA is an appropriate choice as a key anticancer agent to inhibit tumor cell development.

## Molecular targets for natural chemopreventive agents

In the study of cells and animal models, natural products, such as alkaloids, sesquiterpenes lactones, diterpenoids, flavonoids, and polyphenolics, have been widely studied and discovered to exhibit a considerable range of chemopreventive qualities against several forms of cancer. Many other preventives peruse are underway. The pathways of cell signaling, triggered by natural anticancer chemical substances, are various and specific to individual functions for insistence. Besides that, depending on the cell categories, the same substance stimulates multiple signaling pathways (Millimouno et al., 2014).

### p53 Family members

In regulating the genomic integrity, cell cycle, apoptosis, deoxyribonucleic acid repair and response to variations of tumor protein p53 gene and TP53 gene, genotoxic stresses, regarded as the protector of the genome, play an indispensable role (Heinrich et al., 1998; Budram-Mahadeo et al., 2002; Bode and Dong, 2004). Regulation of p53 signaling may suppress the growth and spread of cancer and induce apoptosis of cells (Gong et al., 2017; Su et al., 2017). In GC cells, the signaling pathways which rely on p53 are essential elements to stress cellular responses. Fourcelline in parthenolide, NCI-H1299 lung carcinoma, RKO colon carcinoma, HCT116, and HL60 myeloblastoma activated the substantial decrease in the apoptotic cell frequency in the p53-proficient UV-irradiated row (Guzman et al., 2007; Szoltysek et al., 2008). Parthenolide stimulated p53 and other tumor-suppressor proteins controlled by double mouse minute 2 homolog (MDM2) (Gopal et al., 2009). In PC-3 cells, alanto-lactone causes p53-independent apoptosis in carcinoma of the prostate (Rasul et al., 2013).

### Nuclear factor-Kappa B

A prominent transcription factor is the NF-kB (pro-oncogenic nuclear factor-Kappa B) composed of strongly correlated proteins that form dimers and interact with the kB B site within target gene promoters. It can enhance target gene transcription by recruiting coactivators and corepressors (Aggarwal, 2004). Genes that, after a viral infection, are used to delegate the replication of (a viral gene) through the presence of a gene at another locus are associated with angiogenesis, cell proliferation, apoptosis, metastasis, tumor cell invasion; the NF-kB pathway takes part in an influential role in carcinogenesis (Orlowski and Baldwin, 2002). The NF-kB transcription factor family comprises five types, are- NF-kB1 (p50), NF-kB2 (p52), c-Rel, RelB, and RelA (p65), which express the N-terminal Rel homology domain essential for ankyrin repeat deoxyribonucleic acid (DNA) binding and homodimerization and heterodimerization, encompassing the NF-kBB1 nuclear position series (Aggarwal, 2004; Baud and Karin, 2009).

Nuclear factor-related factor 2 is a possible molecular source that compounds found in nature for cancer prevention. By inducing the procurement of NrF2, some limited natural substances have been identified as possible contenders for chemoprevention. And that is in the nucleus, which actively participates in the transcriptional activation of phase II detoxification enzymes. In the Choi-CK and SCK cells, low parthenolide thresholds resulted in the induction of Nrf2-dependent HO-1, followed by a decrease in its apoptogenic activity. Furthermore, with the protein kinase C-a inhibitor Ro317549 (Ro), parthenolide-mediated apoptosis inhibits activation and nuclear translocation of nuclear factor erythroid 2-related factor 2(Nrf2), resulting in HO-1 expression blockage (Millimouno et al., 2014).

### Activator protein 1

Cellular proliferation, modification, and death have been described in the transcription factor. The target genes and molecular pathways modulating these mechanisms have previously been investigated using mice and cells missing AP-1 components. Appropriately, c-growth-promoting Jun’s function is negotiated by tumor suppressor incarceration and up-regulation in positive cell cycle regulators (Shaulian and Karin, 2002). Other naturally occurring chemopreventive substances have also been shown to decrease AP-1 synthesis and amplify AP-1 target genes, which is ultimately connected with their chemopreventive potential. These substances include resveratrol, green tea, and curcumin. Activator protein 1 (AP-1) transcriptional activity is regulated by green tea polyphenols in a variety of cell types, which is crucial for their function as development inhibitors. A cell employs transcriptional activity to monitor the translation of DNA to RNA and hence organize gene activity (Amin et al., 2009).

### Signal transducers and activators of transcription pathway

STAT (Signal Transducers and Activators of Transcription) is a unique signal transduction pathway to the nucleus discovered in connection with IFNN through the analysis of transcriptional regulation. Several procedures, such as development, immune system feature, multiplicity, differentiation, survival, and EMT (epithelial to mesenchymal transformation), have been involved (Sano et al., 1999; Silver and Montell, 2001). Utilization of different tyrosine kinases leads to STAT protein phosphorylation, nuclear localization, dimerization, linking to and significant transcription of specific DNA components. So many other cancers, namely lymphoma, myeloma, leukemia, and several solid tumors, have been documented to require constitutive STAT3 and STAT5 activation (Rubin Grandis et al., 1998; Sano et al., 1999). Natural substances have been involved in modulating STAT function in tumor cells over the last couple of years. STAT1 dephosphorylation managed to prevent wedelolactone by specifically inhibiting T-cell protein tyrosine phosphatase, an essential enzyme in T-cells for STAT11 tyrosine phosphatase, and STAT1 dephosphorylation inhibited wedelolactone by specifically inhibiting T-cell protein tyrosine phosphatase, which is vital for STAT11 tyrosine phosphatase (Liu et al., 2012; Chen et al., 2013). Parthenolide (C_15_H_20_O_3_ ) shows significant transcriptional repression of proapoptotic genes activated by STAT inhibition, and alantolactone prevents the formation of STAT3 in HepG2 (Liver Hepatocellular Carcinoma) cells (Legendre et al., 2003; Nakshatri et al., 2004; Khan et al., 2013). In addition to validating STAT as a new goal for chemotherapy of cancer, these combined findings including both *in vivo* studies and *in vitro* have also provided the basis for the production of natural component STAT inhibitors (Millimouno et al., 2014).

### Growth factors and their receptors

Factors that foster growth are proteins that associate with the cell surface receptors and documented to influence various cellular mechanisms, with the most important influence of causing apoptosis, proliferation of cells, cytoskeleton rearrangement (Klippel et al., 1996; Kauffmann-Zeh et al., 1997). In carcinogenesis, many growth factors signaling molecules are involved. One of them is a platelet growth factor, endothelial growth factor, the transformation of growth factor, FGF, colony-stimulating factor and insulin-like growth factor (Han et al., 2007). Numerous downstream signals, such as PI3K-Akt and Ras-MAPK, are also involved as a significant intracellular pathway outcome of growth factor receptor stimulation. The critical impact of these signaling pathways is that many natural chemopreventive and chemotherapeutic compounds relate to reduced prognosis and tumor growth, which are becoming aims. Biological agents, specifically the drug selected, quickly induce Akt phosphorylation after activation, which could be used as an active inhibitor of cancer cells (Millimouno et al., 2014).

### Immunoprevention

A re-emergence of concern in cancer immunosurveillance and an expansion of this concept into one termed cancer immunoediting especially have been seen over the past 15 years. The immune system provides protection to the body in the development of primary non-viral cancers and sculpts the immunogenicity of tumors, accompanied by clear observational evidence from murine tumor models and interesting comparative results from human cancer experiments (Dunn et al., 2004). Numerous different natural agents, essential to their prophylactic chemical ability, have modulated specific host factors. Stimulating IL-12-dependent deoxyribonucleic acid (DNA) maintenance, encouraging tumor cell apoptosis, causing cytotoxic (CD8^+^) T cells, and suppressing angiogenic factors have been shown to inhibit UV-induced skin cancer. Epigallocatechin-3-gallate (EGCG) has improved DNA vaccination-affected CD8^+^ T cell-mediated antitumor immunity (Amin et al., 2009). 4,7, 4′-trihydroxyisoflavone (Isoflavone genistein) is an elevated phytoestrogen that has been associated with soy products connected with a low prevalence of breast and prostate cancer. The conceivable immune system outcomes of genistein were monitored in adult female B6C3F1 mice. Gavage or genistein is administered to mouse groups for 28 days (Guo et al., 2001). In a murine xenograft model, VEGF luteolin inhibited vascular-induced angiogenesis and *in vivo* tumor development. A range of research has already shown that the purpose of chemical prevention of Curcumin is angiogenesis (Amin et al., 2009).

## Safety and effectiveness of chemopreventive agents

While the field of cancer chemoprevention is emerging beyond its pioneering phase, widespread acceptance and use have not yet been established. Many agents described above have been identified through studies *in vitro*, animals, and in humans that can inhibit the growth of cancer and other mutation illnesses. These agents should also meet specific requirements, including 1) low price, as defined by standard costing and the target population, 2) pragmatism of use, as determined by accessibility, storage environments, and route of administration, 3) efficacy, and 4) safety (Kelloff et al., 2004; Ferguson et al., 2004; D’Agostini et al., 2005). The Latin word primum non-nocere serves as a reminder that the most important condition for a medical treatment is that it not cause harm to healthy people. Accordingly, the essential need for pharmacological agents employed in the treatment of people living with cancer is their efficacy, even though this kind of treatment is expensive and inconvenient and associated with severe harmful effects (De Flora and Ferguson, 2005). However, certain chemopreventive drugs, which have been demonstrated to be effective but are also toxic, have been described. The effects of chemotherapeutic agents in cancer development are based on *in vitro* studies and animal studies, which typically use higher dosage levels than what is consumed by humans, and it might be challenging to adopt these results directly to humans. Many chemopreventive agents can have side effects, including mutagenicity, carcinogenicity, and other toxic effects (Kelloff et al., 2000; MB S and SuhN., 2000). Therefore, It is thus necessary to develop definite preventative recommendations for distinct types of malignancies that should be carefully designed, considering ethnic differences, efficacy, and safety of chemopreventive medicines in mind (Lee and Park, 2003).

## Conclusion and future perspective

Cancer is perhaps the deadliest disease in the Universe, and it has a massive impact on society, with one out of every three people in the world who have some kind of cancer. Although several existing drugs are ineffective in providing complete cancer protection, it is critical to be developed new treatment methods to treat or slow cancer progression. Bioactive compounds have a wide range of chemical configurations and are likely to be useful in cancer therapy. Anticancer properties vary depending on the variable. Along with their poor aqueous solubility and fast digestion, flavonoids may enhance the performance of stomach, lung, esophageal, colon, and endometrial cancers with limited extreme harmful effects. Alkaloids have a great deal of difficulty meeting their intended destination due to their low bioavailability and poor aqueous solubility. Natural compounds will serve as a stepping stone for reducing the public health effects of primary cancers in the future, thanks to the integration of chemoprevention and chemotherapy drug production. These compounds like chemotherapeutic and immunomodulators are linked to several different targets. Signaling pathways are connected to complex chemical configurations, and this connection is crucial for drug production to continue.

According to data from *in vivo* human and animal research as well as *in vitro* tests, natural products (natural agents) such as curcumin, isoflavones, resveratrol, I3C, DIM, EGCG, and lycopene have inhibitory action on carcinogenesis and cancer progression. Many cells signaling pathways are thought to be involved in these effects, including the NF-B, Wnt, Notch, Akt, MAPK, p53, AR, and ER pathways, among others. Cancer cells constantly exhibit changes in a variety of cellular signaling pathways as a result of the complex interactions between cell signaling networks. As a result, managing cancer cell behavior like cell growth inhibition and death requires medicines that can target numerous cells signaling pathways, and many of these natural products are now regarded to be great examples of natural agents that can target multiple pathways. As a result, we believe that these non-toxic chemicals derived from nature’s bounty could be useful in the prevention and/or treatment of most human cancers, either alone or in combination with established therapies (chemotherapy and radiotherapy). Further in-depth mechanistic research *in vitro*, as well as appropriate and relevant animal model studies *in vivo*, as well as unique clinical trials, are required in the future to fully comprehend the significance of these and other natural products in human health and disease.
